# Cytomorphometric Analysis of Inflammation Dynamics in the Periodontium Following the Use of Fixed Dental Prostheses

**DOI:** 10.3390/molecules25204650

**Published:** 2020-10-12

**Authors:** Artak Heboyan, Azeem Ul Yaqin Syed, Dinesh Rokaya, Paul R. Cooper, Mikael Manrikyan, Marina Markaryan

**Affiliations:** 1 Department of Prosthodontics, Faculty of Stomatology, Yerevan State Medical University, Str. Koryun 2, Yerevan 0025, Armenia; 2 Department of Prosthodontics and Implantology, College of Dentistry, King Faisal University, Al-Hofuf, Al-Ahsa 31982, Saudi Arabia; 3 Department of Clinical Dentistry, Walailak University International College of Dentistry, Walailak University, Bangkok 10400, Thailand; 4 Department of Oral Sciences, University of Otago, Faculty of Dentistry, Sir John Walsh Research Institute, Dunedin 9054, New Zealand; p.cooper@otago.ac.nz; 5 Department of Pediatric Dentistry and Orthodontics, Faculty of Stomatology, Yerevan State Medical University, Str. Koryun 2, Yerevan 0025, Armenia; MikaelA@mail.ru; 6 Department of Therapeutic Stomatology, Faculty of Stomatology, Yerevan State Medical University, Str. Koryun 2, Yerevan 0025, Armenia; marmiga@mail.ru

**Keywords:** periodontium, epithelial cells, polymorphonuclear neutrophils, fixed partial dentures, CAD/CAM, gingival crevicular fluid

## Abstract

Cytomorphometry is used in the sampling of biological materials and diagnostic procedures. The use of cytological studies in periodontal diseases is not well described in the literature. Our study aimed to quantitatively assess the inflammation dynamics using cytomorphometric analysis of the periodontium before and after the use of fixed dental prostheses. Following ethics approval, a total of 105 subjects were divided in 3 groups as gingivitis (*n* = 23), periodontitis (*n* = 58), and healthy periodontium (control) (*n* = 24). The fixed dental prostheses (crowns and fixed partial dentures) were fabricated from cobalt-chrome metal-ceramic prostheses using the conventional method (C/M-CoCr), cobalt-chrome metal-ceramic prostheses by the computer-aided design and computer-aided manufacturing (CAD/CAM) technique (C/C-CoCr), and zirconia-based ceramic prostheses by the CAD/CAM technique (C/C-Zr) among subjects with gingivitis and periodontitis. The gingival crevicular fluid (GCF) was obtained from subjects before and after the use of the prostheses. The total count of epithelial cells and the connective tissue cells or polymorphonuclear neutrophils (PMNs) in GCF were studied using cytomorphometric analysis. The Statistical Package Tor the Social Sciences (SPSS), Version 20 (IBM Company, Chicago, IL, USA) was used to analyze the results and the significance level was set at *p* = 0.05. The data for before and after the use of the prostheses were compared using independent *t*-Tests. Similarly, the results after the use of prostheses in gingivitis, periodontitis, and control in each type of prostheses were compared using One-way ANOVA with post hoc using Scheffe. The total epithelial cells and the PMNs were determined along with the epithelium/leukocyte index. Regardless of the prostheses type used, no significant change in the parameters was identified among patients with a healthy periodontium, before and after prosthetic treatment. In all study groups, a statistically increase (*p* value < 0.05) was observed in the oral epithelial cell counts and a statistically decrease (*p* < 0.05) in the PMNs count following the use of the fixed prostheses. Data on cytomorphometric analysis could enable the selection of the most appropriate prostheses for use in patients with periodontal pathologies. When choosing prostheses, changes in the composition of GCF could be considered as a useful criterion for their use.

## 1. Introduction

Partial edentulousness is one of the most common healthcare problems and occurs in 75% of the population in many regions of the world [1]. In the Republic of Armenia, 63% of young age (range 35–44 years old) and 76.5% of elderly individuals (over 70 years old) present partial edentulousness and need prosthodontic treatment [2].

With the loss of teeth, several significant changes occur, such as impairment of biomechanics in the dentofacial system, unaesthetic, deterioration of periodontal tissues, and negative effects on the general health of the patient [3,4]. Delayed prosthodontic treatment causes complications within the periodontal tissues aggravated by tooth loss [5,6,7]. Prosthodontic restoration following tooth loss with fixed dentures restores the integrity of the dentition. However, if this is not restored appropriately, the prostheses are often accompanied by marginal periodontitis or an unpredictable gingival recession. In the absence of preventive measures, it may lead to loss of the epithelial attachment and endanger biological width [8,9,10]. It has been reported that periodontal disease resulting in tooth loss was the 11th most common disease worldwide [11,12].

It was found that the periodontal factors do not usually have a direct effect on the survival of a fixed prosthesis; the harmony between the prosthesis and the periodontium is important for the esthetics and longevity of the prosthesis [13]. The prosthesis design, the number of abutment teeth and their condition, pontic design, occlusion, and material should be considered for fixed prosthodontic treatment. In addition, the preparation margin, contour, and emergence profile of the prosthesis can influence the gingival tissues response to the prosthesis.

Maintenance of periodontal health at the margins of crowns represents a complex challenge for a prosthodontists and locating the margin of restoration in relation to the alveolar bone height is one of the important factors to ensure the long-term health of the gingiva [14,15]. Crowns and fixed partial dentures may cause interference with the host defenses to create areas of microbial contamination leading to bacterial biofilm formation and subsequent damage to the periodontium. [16].

Al-Sinaidi and Preethanath [17] assessed the periodontal status of fixed prostheses, and the effects of sub- and supra-gingivally placed crown margins were also assessed. It was found that the abutment teeth with higher plaque and gingival indices and greater probing pocket depth than non-abutment teeth. In addition, the abutment teeth with higher mean values for clinical parameters were present in subjects who were older than 46-years of age and those who had their functioning fixed prostheses for >5 years. The teeth with supra-gingivally placed crown margins had significantly higher mean values of plaque index, gingival index, and probing pocket depth than teeth with sub-gingival crown margins. Additionally, the individual’s age, duration of insertion of fixed partial dentures, and location of the crown margins affect the periodontal health of the abutments.

Several studies have been conducted to determine the cytological composition of gingival crevicular fluid (GCF) in inflammatory diseases of periodontal tissues for diagnostic purposes [18,19,20,21]. However, there remain no comparative data on the change in GCF parameters during prosthodontic treatment using fixed prosthesis fabricated from various materials [22]. Without due intervention, inflammatory changes in the periodontium might lead to further tissue destruction resulting in early tooth loss [23,24,25]. Pathological progression can occur due to oral keratinocyte responses to the presence of periodontal infection foci [26,27,28]. In healthy tissues, these cells are constantly shed and replaced by basal progenitors within the gingival sulcus. Notably, this rate of turnover increases as the disease progresses [29,30,31]. Furthermore, in the gingival pockets of patients with periodontal disease, polymorphonuclear leukocytes (PMNs) are abundant. It has been shown that 47% of the cells found in the gingival sulcus were white blood cells, with 98% of these leukocytes being PMNs. Notably, the absolute number of cells increased in proportion to inflammation severity, while the differential number of PMNs was 95–97%, lymphocytes 1–2%, and mononuclear cells represented 2–3% [18,32]. Cytomorphometry is routinely used in several diagnostic procedures [33]. This approach is relatively rapid, straightforward, and reliable, with exfoliative cytology allowing for repeated sampling of biological material without affecting the integrity of the local host tissue. Consequently, this approach can be performed recurrently in screening programs and during general dental examinations [34]. However, the use of cytological studies in periodontal diseases is not well described in the scientific literature and appears rarely used in dental and periodontal practice [35,36].

Many studies have sought to identify improvements for prosthodontic treatment outcomes among patients to decrease complications and drawbacks associated with current practices [37]. Therefore, our study aimed to quantitatively assess the inflammation dynamics using cytomorphometric analysis of the periodontium before and after the use of fixed dental prostheses; cobalt-chrome metal-ceramic prostheses by the conventional method (C/M-CoCr), cobalt-chrome metal-ceramic prostheses by CAD/CAM technique (C/C-CoCr), and zirconia-based ceramic prostheses by CAD/CAM technique (C/C-Zr). Periodontal status was assessed using the clinical signs of inflammation and the Community Periodontal Index (CPI) score. The results from this study could help to assimilate better choice of prosthetic prostheses to prevent inflammation and destruction of the periodontium.

## 2. Results

### 2.1. Study Subjects (Gingivitis, Periodontitis, and Healthy Subjects)

**Figure 1:**
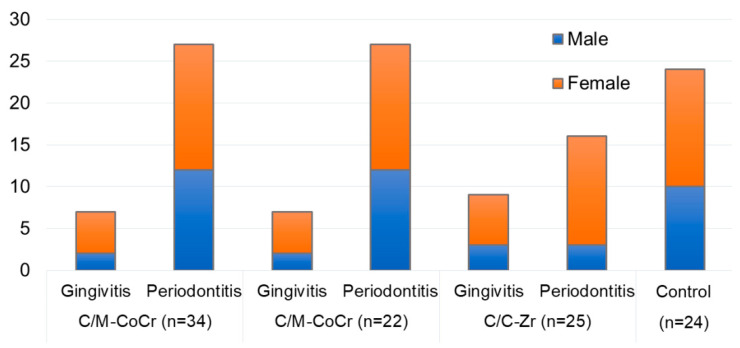
Distribution of patients in the three groups with fixed prostheses in gingivitis and periodontitis, and the healthy (control) group. The study included C/M-CoCr = Cobalt-chrome metal-ceramic prostheses by the conventional method; C/C-CoCr = Cobalt-chrome metal-ceramic prostheses by CAD/CAM technique; C/C-Zr = zirconia-based ceramic prostheses by the computer-aided design and computer-aided manufacturing (CAD/CAM) technique.

The distribution of the patients with gingivitis and periodontitis in three groups with different types of fixed prostheses is shown in Figure 1. The number of subjects with gingivitis was 7 (18.9%) in C/M-CoCr, 9 (27.3%) in C/C-CoCr, and 7 (20%) in C/C-Zr groups. Similarly, the number of subjects with periodontitis were 27 (27.3%) in C/M-CoCr, 16 (48.5%) in C/C-CoCr, and 15 (42.9%) in C/C-Zr groups. In all, a total of 23 subjects had gingivitis and 58 subjects had periodontitis.

### 2.2. Oral Epithelial Cells and Connective Tissue Cells Analysis

The results of the oral epithelial cell and connective tissue cell count performed among patients with gingivitis, periodontitis, and healthy subjects are shown in Table 1. In all groups, a statistical increase (*p* value < 0.05) in the oral epithelial cell counts and a statistical decrease (*p* < 0.05) in the PMN count was observed following the use of the fixed prostheses. Oral epithelial cell numbers considerably predominated over those of PMNs.

### 2.3. Epithelium/Leukocyte Index

**Figure 2:**
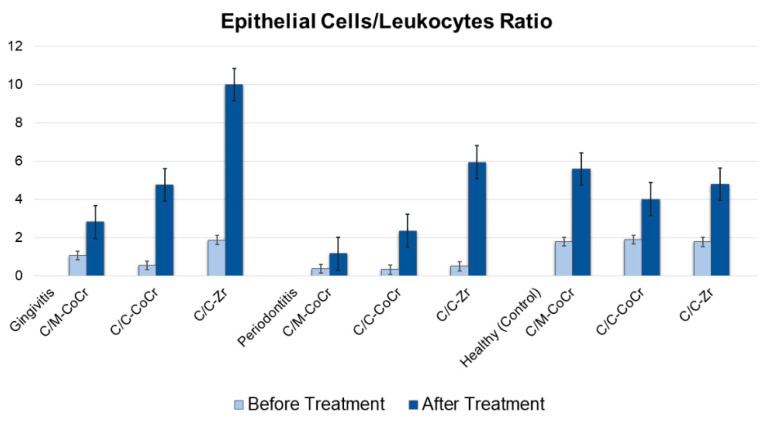
The mean epithelial cells/leukocytes ratios in gingivitis, periodontitis, and healthy (control) subjects. C/M-CoCr = Cobalt-chrome metal-ceramic prostheses by the conventional method; C/C-CoCr = Cobalt-chrome metal-ceramic prostheses by CAD/CAM technique and C/C-Zr = zirconia-based ceramic prostheses by CAD/CAM technique.

The results of the epithelial cells/leukocytes ratio are shown in Figure 2. The mean epithelial cells/leukocyte index improved considerably in all groups of patients with gingivitis, periodontitis, and healthy subjects.

The comparison of the dynamics of cytomorphometric indicators in three fixed prostheses among patients with gingivitis, periodontitis, and healthy subjects before and after treatment is shown in Table 2. In C/M-CoCr vs. C/C-CoCr, a significant difference was found for the epithelial cells in healthy (before treatment) and in gingivitis, periodontitis (after treatment), and a significant difference was found for the PMNs in gingivitis (after treatment) and in gingivitis and periodontitis (after treatment). Similarly, in C/M-CoCr vs. C/C-Zr, a significant difference was found for the epithelial cells in healthy subjects (before treatment) and in all groups (after treatment), and significant difference was found for the PMNs in periodontitis (before treatment), and in gingivitis and periodontitis (after treatment). Finally, in C/C-CoCr vs. C/C-Zr, a significant difference was found for epithelial cells in all groups (before treatment), and a significant difference was found for the PMNs in all groups (before treatment) and in gingivitis and periodontitis (after treatment).

## 3. Discussion

The present study identified oral epithelial changes using exfoliative cytology through cytomorphometry and morphological methods in gingivitis and periodontitis and compared them with healthy individuals. The cytomorphometric data obtained from the GCF samples made it possible to identify the cellular composition within the periodontal pockets among partial edentulousness patients. Changes in periodontal tissues taking place during restoration with fixed dental prostheses occur as stimulation of regenerative and adaptive processes. Potentially these changes could be used prognostically to enable positive outcomes for patients when choosing prosthetic prostheses and further prosthodontic treatment. Both qualitative and quantitative changes were revealed in the cellular composition of the GCF among patients with inflammatory periodontal diseases using the community CPI and clinical signs of inflammation (discomfort, tenderness of the gums, bleeding with different levels of severity, and bad breath). Patients with gingivitis present red and swollen gingiva and bleeding on probing, whereas patients with periodontitis present swollen hyperemic gums, which bled on probing as well as the presence of soft dental plaque of varying degrees, supra- and subgingival calculus and periodontal pockets of a range of depths.

In this study, using microscopic examination of GCF, the main components of the cellular scatter of gingival cytograms comprised flat epithelial cells and the gingival immuno-competent cells of PMNs. The presence of these cells was noted both among healthy subjects and among those with periodontal diseases. Indeed, smear samples obtained from the subjects with healthy periodontium, PMNs, and epithelial cells were detected. With the occurrence of the inflammatory process in the periodontium, the count of PMNs, which are reportedly destructive for periodontal tissues, was observed to increase among subjects from all groups. Examination of the GCF samples revealed cytomorphologic differences that were potentially associated with the manufacturing technique and the material used in the dental prostheses. A relatively high count of PMNs in GCF smears suggested an increase in vascular permeability due to exposure to periodontal bacteria as well as the presence of an inflammatory process among the subjects of the C/C-CoCr group with periodontitis, which also proceeded with a decrease in oral epithelial cell count. In this group, the epithelium/leukocyte index was 0.32. One year after prosthodontic treatment with fixed dental prostheses, the periodontal status was noted to improve significantly according to cytomorphometric indicators. The epithelium/leukocyte index increased to 2.35. In GCF smears obtained from the gingival sulcus of subjects in the C/C-Zr group with periodontitis, the immunocompetent cell count was relatively high. The epithelium/leukocyte index before treatment was 0.499. The positive dynamics among patients with periodontitis was confirmed by the cytological data in the GCF derived from the periodontal pockets. Cytological analysis among the subjects from this group with periodontitis revealed the cellular composition of the GCF to be similar to that from healthy periodontium and the epithelium/leukocyte index was increased to 5.94. These data suggested a limiting of the inflammatory process in the periodontium (Figure 2).

In this study, the mean epithelium/leukocyte index improved considerably in all groups of patients with gingivitis. Thus, in the C/M-CoCr group, it increased from 1.05 to 2.8, while in the C/C-CoCr and the C/C-Zr groups, it increased from 0.54 to 4.7 and from 1.86 to 9.98, respectively. The reduced epithelial cells/leukocyte index before the treatment was most likely due to a result of the inflammatory reactions occurring in the periodontal tissues. The change in leukocyte-epithelial cell ratios correlated well with the clinical manifestations of the disease. Indeed, as the disease progresses, PMNs provide the first line of defense, and these characteristic changes were observed in the GCF cytograms. The analysis of the data on the cellular composition of the GCF among the subjects of the control group before prosthodontic treatment indicated that the PMN count increased as the inflammation in the periodontium became more severe. However, the epithelial cell count was reduced almost three times in the periodontitis group. At the same time, one year after prosthodontic treatment, along with a decrease in the PMNs count, an increase in oral epithelial cell count as well as an increase in the epithelium/leukocyte index from 0.36 to 1.16, was observed. The cell ratio change in the leukocyte-epithelial formula agreed with the clinical manifestations of the disease, reported in some scientific papers [28].

However, in this study, regardless of the type of prostheses used, no considerable parameter changes were observed among the patients with a healthy periodontium before and after prosthodontic treatment. The analysis of the comparative results of cytomorphometric indicators before and after 12 months of the restoration in the group of patients with gingivitis was in agreement. Significantly, similar epithelial cell count, as well as an increased PMN count, was observed in all groups before treatment. No statistical difference was found among the groups according to these indicators (*p* > 0.1). One year after prosthetic treatment, an increase in the epithelial cell count was detected in all groups and a significant decrease in the PMNs count was observed compared with the data obtained before treatment onset. Among the patients in the C/C-Zr group, the epithelial cell count was 1.33 times greater (*p* < 0.01), and the leukocyte count was 2.7 times less (*p* > 0.5), without statistically significant differences found between the indicators of the C/C-CoCr group and C/C-Zr group (*p* > 0.1).

The results obtained revealed no significant change in the cytomorphometric parameters among patients with a healthy periodontium before and after acquiring prosthesis, regardless of the prosthesis type. The data were consistent with studies conducted by other authors [36,37].

As shown by the results of our study, PMNs were present in fixing all types of prosthodontic prostheses, but their quantitative composition was different. Improved cytomorphometric indicators were observed among patients with periodontal diseases, depending on the material of prostheses. The results revealed improvement among the patients in the C/C-Zr group compared with the C group, which was also confirmed by several studies [20,22].

Finally, the results indicated that a procedure could be developed and used to justify the selection of a prosthesis, which would be appropriate for the patient and prevent the progression of inflammatory and destructive processes within the periodontium. Following the completion of the study, the subjects with gingivitis and periodontitis were treated by a prosthodontist and periodontist and followed up for 6 months. Hence the subjects were followed up 6 and 12 months after the treatment.

A fixed prosthesis may compromise the periodontal health of the mucosa if pontic hygiene is not maintained by the removal of plaque. Hence, the patients’ compliance in maintaining adequate oral hygiene is, therefore, essential for the longevity of the prosthesis [13,38]. The limitations of this study were due to the time factor, as recruiting more patients was difficult. The small sample size may have negatively impacted on statistical significance differences. In this study, we did not evaluate the correlation between the stages of periodontitis and various fixed prostheses factors. Work could be extended in the future to investigate the correlation of stages of periodontitis and various prosthetic factors (occlusal status, margin design, and pontic design). In addition, this study could be undertaken to include a larger sample size by including both quantitative and qualitative assessment studying the type of immunomodulatory effects.

## 4. Materials and Methods

### 4.1. Ethics Approval and Consent to Participate

Patients’ rights were thoroughly explained to the participants, including the right to remove themselves from the study without any further explanation being required. Following the Declaration of Helsinki, before treatment began, all participants signed forms giving consent, confirmed by the Ethics Committee of Yerevan State Medical University (IRB APPROVALN12-5/2019, 13 June 2019). All the ethical principles described in the Helsinki Declaration were adhered to throughout this study.

### 4.2. Patients Inclusion and Exclusion Criteria

A total of 105 patients aged 19 to 72 years old, who underwent complex prosthodontic treatment, were examined during dental appointments at Keshishyan, Nord KS and Medesy Esthetic Dental Clinics in Yerevan, Armenia. The selection criteria of the study subjects in the study are shown in Table 3.

### 4.3. Prosthetic Fabrication and Treatment Groups

Patients were divided into three groups according to the type of fixed dental prostheses (crowns and fixed partial dentures); cobalt-chromium metal-ceramic prostheses made by conventional techniques (CM-CoCr group, 37 patients), cobalt-chromium metal-ceramic prostheses made by CAD/CAM technology (C/C-CoCr group, 33), and zirconia-based ceramic prostheses made by CAD/CAM technology (C/C-Zr group, 35 patients). For the C/M-CoCr group, at first, wax copings were made, and these copings were replaced by the metal copings using the lost-wax technique, and finally, metal-ceramic prostheses were fabricated. For the C/C-CoCr group, the cobalt-chromium metal-ceramic prostheses were made by CAD/CAM technology using Ceramill Sintron Technology. The copings were milled from soft pre-sintered cobalt-chromium alloy by CAD/CAM and then sintered under high pressure. For the C/C-Zr group, the zirconia-based ceramics were made by CAD/CAM technology. At first, Zircon cores were milled by CAD/CAM technology from pre-sintered zirconia blocks. These prostheses were then sintered, and porcelain layers were applied.

### 4.4. Study Groups

During the study, each group was subdivided into three further subgroups in accordance with accompanying disease, i.e., subjects with gingivitis (23 patients), periodontitis (58 patients), and healthy subjects with no gingivitis or periodontium (24 subjects). The GCF was studied before and six months after the delivery of the prostheses among subjects with gingivitis, with periodontitis, and healthy periodontium (control). The patients maintained hygiene from the diagnosis until the GCF sample collection.

The gingivitis and periodontal diagnoses were made by two calibrated examiners using the CPI recorded for each sextant [39]. Ten index teeth (17, 16, 11, 26, and 27 in the maxilla and 31, 36, and 37, 46, and 47 in the mandible) in six sextants (17–14, 13–23, 24–27, 37–34, 33–43, and 44–47) were probed, and scores were assigned to each sextant on the following basis as shown in Table 4.

The group of patients with gingivitis included subjects with codes 1 and 2 by the CPI; the group of patients with periodontitis included subjects with codes 3 and 4, while the CPI code was 0 among patients with healthy periodontium.

Following the completion of the study, the subjects with gingivitis and periodontitis were treated by prosthodontist and periodontist and followed up for six months. Hence in this study, the subjects were followed up 6 and 12 months after the treatment.

### 4.5. Cytological Sampling and Analyses

The GCF from the gingival sulcus or periodontal pocket was obtained before treatment and six months after delivery of the prostheses from subjects with gingivitis, periodontitis, and healthy subjects (no gingivitis or periodontitis). For the GCF collection and cytological sampling, at first, the cytological preparations were done. The area was dried by air spray and cotton rolls. The tooth selected was isolated, and the absorbent paper strip (Periopaper, Ora flow Inc., Smithtown, NY, USA) was placed in the gingival crevice using a forceps. The strip was inserted ay the bottom of each pocket (~1 mm) in the gingival crevice, placed for 30 sec and then removed [40,41]. Then, the strips were placed in a transportation vial and sent to the laboratory immediately using Hicultur Transport swab Himedia in Amies Charcoal medium (Thermo Fisher Scientific, Invitrogen BioServices India Pvt Ltd., Mumbai, India).

At the laboratory, the strips were placed on a sterile and grease-free dry glass slides, then the oral exfoliative cytology was done as mentioned by Castro et al. [18]. The epithelial cells, connective tissue cells, and polymorphonuclear neutrophils (PMNs) from the GCF were studied by cytomorphometric analysis. Smears were fixed in methanol for 2 to 3 min, then dried, and stained using the Diff-Quick technique. The Romanowski group of stains are defined as being the black precipitate formed by the addition of aqueous solutions of methylene blue and eosin, dissolved in methanol. The stained glass was originally designed to incorporate cytoplasmic (pink) staining with nuclear (blue) staining and fixation as a single step for smears and thin films of tissue (spread preparations of omentum) [42,43].

Samples were observed under a PrimoStar microscope (Carl Zeiss Microscopy GmbH, Jena, Germany) in the Cytology Department of Davidyants Laboratories (GYSANE Limited Liability Company, Yerevan, Armenia), and the total count of epithelial cells and PMNs were recorded. In addition, the ratio of a total count of epithelial cells and the connective tissue cells (polymorphonuclear neutrophils) were determined along with the epithelium/leukocyte index. The tests were performed by one staff expert in the histology.

### 4.6. Statistical Analysis

Descriptive statistics were performed using the Statistical Package Tor the Social Sciences (SPSS), Version 20 (IBM Company, Chicago, IL, USA). The data obtained before and after the use of the prostheses were compared using Independent *t*-Test. Similarly, the data obtained following the use of prostheses in gingivitis, periodontitis, and control, in each type of prostheses, were compared using One-way ANOVA with Post-hoc using Tukey. The significant level was set at *p* = 0.05.

## 5. Conclusions

Regardless of the prostheses type (C/M-CoCr, C/C-CoCr, and C/C-Zr), no significant change in the parameters was identified among patients with a healthy periodontium, before and after prosthetic treatment. In all study groups (gingivitis, periodontitis, and healthy), a statistical increase was observed in the oral epithelial cell counts, and a statistically decrease in the PMNs count following the use of the fixed prostheses. A close relationship exists between the cellular and humoral immunities of periodontal pockets, mainly with PMNs playing a key role in the inflammatory processes. The cytological method provides an informative assay that allowed for the identification of etiological risk factors for the occurrence of inflammatory periodontal diseases as it reveals the dynamics of the disease during its progression in prosthodontic treatment. This approach provides an opportunity for personalized medicine and the development of new diagnostic possibilities.

## Figures and Tables

**Table 1:** Comparison of the dynamics of cytomorphometric indicators among patients with gingivitis, periodontitis, and healthy subjects with different fixed prostheses.

Subgroup	Cells Measurements	Groups		*p* Value
		Before Prostheses (Mean ± SD)	After Prostheses (Mean ± SD)	
**Gingivitis Group (*n* = 23)**				
C/M-CoCr	Epithelial cells	11.14 ± 3.4	14 ± 2.2	0.002 *
	PMNs	10.57 ± 4.7	5 ± 1.9	0.005 *
C/C-CoCr	Epithelial cells	9 ± 2.6	16.89 ± 1.8	<0.0001 *
	PMNs	16.56 ± 11.4	3.56 ± 1.6	0.006 *
C/C-Zr	Epithelial cells	11.71 ± 4.2	18.57 ± 3.2	<0.0001 *
	PMNs	6.29 ± 3.2	1.86 ± 0.04	<0.0001 *
**Periodontitis Group (*n* = 58)**				
C/M-CoCr	Epithelial cells	8.48 ± 3.03	11.67 ± 2.7	<0.0001 *
	PMNs	23.33 ± 10.9	10.07 ± 3.4	<0.0001 *
C/C-CoCr	Epithelial cells	7.25 ± 2.8	14.12 ± 2.7	<0.0001 *
	PMNs	22.63 ± 11.7	6.0 ± 2.2	<0.0001 *
C/C-Zr	Epithelial cells	9.13 ± 2.9	14.67 ± 3.02	<0.0001 *
	PMNs	18.27 ± 6.2	2.47 ± 0.9	<0.0001 *
**Control Group (*n* = 24)**				
C/M-CoCr	Epithelial cells	13.67 ± 1.52	18.67 ± 3.05	<0.0001 *
	PMNs	7.67 ± 2.51	3.33 ± 1.88	<0.0001 *
C/C-CoCr	Epithelial cells	16.12 ± 4.67	16.75 ± 4.23	0.006 *
	PMNs	8.50 ± 2.04	4 ± 2.97	0.04 *
C/C-Zr	Epithelial cells	11.08 ± 2.39	14.77 ± 2.92	<0.0001 *
	PMNs	6.23 ± 2.5	3.08 ± 1.09	0.006 *

C/M-CoCr = Cobalt-chrome metal-ceramic prostheses by the conventional method; C/C-CoCr = Cobalt-chrome metal-ceramic prostheses by CAD/CAM technique; C/C-Zr = Zirconia-based ceramic prostheses by CAD/CAM technique; PMNs = Polymorphonuclear leukocytes; SD = Standard Deviation * Significant at *p* < 0.05.

**Table 2:** Comparison of the dynamics of cytomorphometric indicators in the three fixed prostheses groups among patients with gingivitis, periodontitis, and healthy subjects, before and after treatment.

Group	Cells	Multiple Comparisons		
		C/M-CoCr vs. C/C-CoCr	C/M-CoCr vs. C/C-Zr	C/C-CoCr vs. C/C-Zr
**Before Treatment**				
Gingivitis	Epithelial cells	0.098	0.84	0.026 *
	PMNs	0.020 *	0.126	<0.0001 *
Periodontitis	Epithelial cells	0.062	0.453	0.0018 *
	PMNs	0.923	0.017 *	0.049 *
Healthy Subjects	Epithelial cells	0.023 *	0.015 *	<0.0001 *
	PMNs	0.438	0.094	0.003 *
**After Treatment**				
Gingivitis	Epithelial cells	0.0005 *	<0.0001 *	0.062
	PMNs	0.0032 *	<0.0001 *	0.004 *
Periodontitis	Epithelial cells	<0.0001 *	<0.0001 *	0.544
	PMNs	<0.0001 *	<0.0001 *	<0.0001 *
Healthy	Epithelial cells	0.138	0.0006 *	0.122
	PMNs	0.532	0.9058	0.297

C/M-CoCr = Cobalt-chrome metal-ceramic prostheses by conventional method; C/C-CoCr = Cobalt-chrome metal-ceramic prostheses by CAD/CAM technique; C/C-Zr = Zirconia-based ceramic prostheses by CAD/CAM technique; PMNs = Polymorphonuclear leukocytes; SD = Standard Deviation. Multiple comparisons were analyzed using One-way ANOVA with Post-hoc using Tukey. * Significance difference at *p* < 0.05.

**Table 3:** Selection criteria of the study subjects in the study.

Inclusion Criteria	Exclusion Criteria
Patient willing to participate in the study Healthy subjects without underlying systemic diseases Not under any medication Subjects with gingivitis and periodontitis with no other oral diseases (study subjects) and healthy subjects (control) Patients with partial edentulousness and/or with tooth crown destruction	Pregnancy Systemic diseases Surgical and nonsurgical periodontal therapy Patients using antibiotics or anti-inflammatory agents, all within 6 months before their inclusion in the study Smokers

**Table 4:** Clinical assessment of periodontal status carried out by Community Periodontal Index [40].

Score	Sign
Score 0	no signs of disease
Score 1	gingival bleeding after gentle probing
Score 2	presence of supra- or subgingival calculus or other plaque retentive factors
Score 3	4- or 5-mm periodontal pockets
Score 4	6-mm or deeper periodontal pockets
